# Exenatide once weekly improved glycaemic control, cardiometabolic risk factors and a composite index of an HbA1c < 7%, without weight gain or hypoglycaemia, over 52 weeks

**DOI:** 10.1111/dom.12026

**Published:** 2012-11-12

**Authors:** R M Bergenstal, Y Li, T K Booker Porter, C Weaver, J Han

**Affiliations:** 1 International Diabetes CenterMinneapolis, MN, USA; 2 Amylin Pharmaceuticals, LLCSan Diego, CA, USA

**Keywords:** cardiovascular disease, exenatide, GLP-1, glycaemic control, type 2 diabetes

## Abstract

**Aims:**

Type 2 diabetes mellitus (T2DM) is often associated with cardiovascular (CV) risk factors such as obesity, hypertension and dyslipidemia. The objective of this analysis was to evaluate potential effects of exenatide once weekly (ExQW), a GLP-1 receptor agonist, on glycaemic control and CV risk factors.

**Methods:**

This analysis included 675 Intent-to-Treat patients with T2DM [baseline (mean ± SD) HbA1c, 8.1 ± 1.2%; fasting blood glucose (FBG), 166 ± 48 mg/dl; weight, 94.3 ± 19.4 kg; systolic/diastolic blood pressure (SBP/DBP), 129 ± 15/78 ± 9 mm Hg; total cholesterol, 178.5 ± 41.9 mg/dl; low-density lipoprotein (LDL), 100.1 ± 35.0 mg/dl; high-density lipoprotein (HDL), 44.5 ± 11.6 mg/dl; triglycerides, 155.6 ± 3.3 mg/dl; alanine aminotransferase (ALT), 32.1 ± 19.5 U/l] treated with diet and exercise alone or in combination with metformin, sulfonylurea, and/or thiazolidinedione who received 52 weeks of ExQW in four clinical trials.

**Results:**

At 52 weeks, ExQW significantly improved HbA1c [mean (SE) change from baseline, −1.3 (0.05)%], FBG [−36.3 (2.02) mg/dl], body weight [−2.6 (0.19) kg], SBP/DBP [−3.6 (0.56) mm Hg/−1.2 (0.34) mm Hg], total cholesterol, −4.4 (1.33) mg/dl; LDL, −2.6 (1.08) mg/dl; HDL, 1.1 (0.31) mg/dl; triglycerides, −7 (1.6)%], and ALT [−4.3 (0.71) IU/l] concentrations, with greater improvements in patients with elevated analyte levels at baseline. Improvements were observed across a range of background antihyperglycaemia therapies. Of patients completing 52 weeks, 19% achieved the composite American Diabetes Association goal (HbA1c < 7.0%, BP < 130/80 mm Hg, LDL < 100 mg/dl), compared to 1% at baseline. Nearly half (48%) achieved HbA1c < 7.0% without weight gain or major/minor hypoglycaemia. Nausea was the most frequent adverse event and was predominantly mild. Hypoglycaemia was infrequent, and more common with a sulfonylurea.

**Conclusions:**

With 52 weeks of ExQW, patients experienced sustained improvements in glycaemic control and CV risk factors, with an increased likelihood of achieving both a clinically relevant composite outcome (HbA1c < 7% without weight gain or increased risk of hypoglycaemia) and a composite of key therapeutic goals (HbA1c < 7%, BP < 130/80 mm Hg, LDL < 100 mg/dl).

## Introduction

Patients with type 2 diabetes mellitus (T2DM) often exhibit a number of interrelated comorbidities, including obesity, hypertension and dyslipidemia associated with an increased probability of cardiovascular (CV) disease. To encourage more comprehensive treatment, the American Diabetes Association (ADA) established goals (HbA1c < 7.0%, blood pressure <130/80 mm Hg, low-density lipoprotein [LDL] cholesterol <100 mg/dl) to reduce the impact of comorbidities associated with T2DM [1]. Despite significant evidence suggesting improved morbidity and mortality when these goals are met and maintained [1], only one in eight patients in the United States reach the composite ADA goal [2].

Although diet and exercise intervention can improve hyperglycaemia and the comorbidities associated with T2DM, most patients eventually require pharmacotherapy to control hyperglycaemia. Few antihyperglycaemia agents, however, are associated with improvements in interrelated comorbidities and many are associated with weight gain, which may exacerbate hypertension and dyslipidemia. Thus, additional therapy is often needed to treat associated comorbidities. Treatment is further complicated by concerns regarding hypoglycaemia. Another composite outcome (HbA1c < 7.0%, no weight gain and no episodes of hypoglycaemia) has been proposed to improve glycaemic control without increased CV risk from further weight gain or loss of patient compliance because of hypoglycaemia [3].

Exenatide, a glucagon-like peptide-1 receptor agonist (GLP-1RA), has been shown to improve glycaemic control in patients with T2DM through multiple mechanisms of action including increased glucose-dependent insulin secretion, attenuated postprandial glucagon secretion, slowed gastric emptying and increased satiety [4]. Exenatide has also been shown to improve markers of CV risk (body weight, blood pressure and lipids) and hepatic dysfunction (alanine aminotransferase [ALT]), with effects sustained with 3 years of treatment [5]. Of note, due to its glucose-dependent mechanism of action, exenatide treatment is not associated with increased risk of hypoglycaemia [6].

Exenatide is approved for T2DM therapy in a twice daily (ExBID) formulation and in a recently approved once-weekly (ExQW) formulation that provides sustained exenatide concentrations that result in greater 24-h glucose control. Similar to ExBID, improvements in glycaemic control, body weight and CV risk markers have been observed with 24–30 weeks of ExQW treatment [7–9]. As previous ExBID studies suggested that CV risk factors continued to improve with longer exenatide exposures [5,10], we investigated the effects of 52 weeks of ExQW treatment on glycaemic control, body weight, CV risk factors, hypoglycaemia incidence, the impact of background antihyperglycaemia therapies, and the percentage of patients achieving composite treatment outcomes.

## Materials and Methods

### Design Overview

Data were analyzed from 675 Intent-to-Treat (ITT) patients with T2DM treated with background therapies of diet and exercise, metformin, sulfonylurea (SU), thiazolidinedione (TZD) or a combination of agents that received 48–52 weeks of ExQW (2 mg). Patients participated in one of four trials,^a^ including three comparator-controlled trials that showed significantly greater improvement in HbA1c with ExQW compared to ExBID, sitagliptin, pioglitazone and insulin glargine during the 26–30 week controlled periods [7–9] with patients then continuing into open-label extensions to at least 52 weeks, and 1 uncontrolled trial that showed the safety of ExQW for 52 weeks in patients using TZD ± metformin [7–9,11]. For the comparator-controlled trials, only patients who received ExQW treatment during the initial controlled periods and who continued ExQW into the extension periods to 52 weeks were included in this analysis.

Clinical protocols were approved for each site by the appropriate Institutional Review Board. Patients provided written informed consent prior to participation. Studies were conducted in accordance with the principles of the Declaration of Helsinki (1964) up to and including the Seoul revision (2008) 12.

Plasma analytes and HbA1c were quantitated by Quintiles Laboratories (Smyrna, GA; Marietta, GA; Mumbai, India; Livingston, Scotland; Singapore; Pretoria, South Africa) using standard methods. HbA1c was measured using high-performance liquid chromatography methodology.

### Statistical Analysis

The ITT population comprised all randomized patients who received at least one injection of ExQW. The Completer population consisted of ITT patients who completed study visits to at least Week 48 in compliance with the protocols. Descriptive statistics on baseline demographics, and the change from baseline for primary glycaemic endpoints, body weight, blood pressure, lipid and ALT concentrations and concomitant medications were assessed for the ITT and Completer populations. Descriptive statistics for safety analyses were assessed for the ITT population. The percentages of patients achieving treatment goals were evaluated for the Completer population. The correlation between the change in body weight and the change in other parameters was estimated using the Pearson correlation coefficient (r). Missing data for efficacy endpoints were imputed by the last observation carried forward (LOCF) method. Data on mean change from baseline were expressed as mean (SE). Triglyceride data were analyzed in logarithmic scale and geometric mean changes from baseline were provided.

Within-group comparison was conducted using the paired t-test at a 2-sided significance level of 0.05. Incidence of hypoglycaemia events were compared between concomitant SU users and non-SU users using Fisher's exact test. Statistical analysis was performed using SAS (9.2; SAS Institute, Cary, NC, USA).

Treatment-emergent adverse events (TEAEs) were defined as those occurring or worsening during or after the first injection of ExQW. Hypoglycaemia was categorized as major if the event: (i) in the judgment of the investigator or physician, resulted in a loss of consciousness, seizure or coma and resolved after administration of glucagon or glucose; or (ii) required third-party assistance to resolve and had a glucose value of <54 mg/dl. Minor hypoglycaemia was defined as symptoms consistent with hypoglycaemia and a glucose value of <54 mg/dl prior to treatment of the episode.

This posthoc analysis was sponsored by Amylin Pharmaceuticals, LLC. Amylin and Eli Lilly and Company were clinical study sponsors and involved in study design, protocol development and collection, review and analysis of data. Dr. Bergenstal had full access to primary data and led decisions on content.

## Results

### Patient Disposition and Baseline Characteristics

Patient disposition and baseline demographics for the individual studies were described previously [7–9,11]. A total of 141 (21%) patients withdrew (Table 1), resulting in 534 patients (79% of the ITT population) completing 52 weeks of treatment. The most common reasons for withdrawal were patient decision (7.0%), adverse event (5.9%), and loss to follow-up (2.7%). Demographic characteristics of ITT and Completer populations were similar (Table 1).

**Table 1:** Patient disposition and baseline demographics

Disposition		
Intent-to-Treat, N	675	
Completed, n	534	
Withdrew, n	141	
Patient decision	47	
Adverse event	40	
Lost to follow-up	18	
Protocol violation	9	
Did not enter extension period	9	
Entry criteria not met	7	
Investigator decision	5	
Administrative	3	
Loss of glucose control	2	
Sponsor decision	1	

Demographics	ITT	Completer
N	675	534
Gender, male/female (%)	54/46	55/45
Age at consent (years)	55 ± 10.04	55 ± 9.8
Race/ethnicity – n (%)		
Caucasian	478 (71)	394 (74)
Black	41 (6)	27 (5)
Asian	54 (8)	41 (8)
Hispanic	94 (14)	66 (12)
American Indian or Alaska native	6 (1)	6 (1)
Body weight (kg)	94.3 ± 19.4	95.2 ± 19.5
Body mass index (kg/m^2^)	33.1 ± 5.2	33.4 ± 5.3
HbA1c (%)	8.1 ± 1.2	8.1 ± 1.2
HbA1c stratum		
<9.0%, n (%)	512 (75.9)	417 (78.1)
HbA1c (%)	7.6 ± 0.7	7.6 ± 0.7
≥9.0%, n (%)	162 (24.0)	117 (21.9)
HbA1c (%)	9.9 ± 0.6	9.8 ± 0.6
Fasting blood glucose (mg/dl)	166 ± 48	164 ± 46
Duration of diabetes (years)	7 ± 6	7 ± 6
Blood pressure		
Systolic blood pressure (mm Hg)	129 ± 15	130 ± 15
Diastolic blood pressure (mm Hg)	78 ± 9	78 ± 9
Lipids		
Total cholesterol (mg/dl)	178.5 ± 41.9	179.0 ± 42.0
Low-density lipoprotein (mg/dl)	100.1 ± 35.0	100.3 ± 35.3
High-density lipoprotein (mg/dl)	44.5 ± 11.6	44.5 ± 11.5
Triglycerides (mg/dl)*	155.6 ± 3.3	157.0 ± 3.6
Alanine aminotransferase (U/l)	32.1 ± 19.5	32.1 ± 19.3
Diabetes management at screening		
Diet/exercise only	20	17
Metformin only	366	273
Sulfonylurea only	6	5
Thiazolidinedione only	17	16
Metformin + Thiazolidinedione	132	115
Sulfonylurea + other oral antihyperglycaemia agents	126	104

Data are presented as mean ± standard deviation.

* Triglyceride data are presented as geometric mean ± SE.

### Effects on Glycaemic Control and Body Weight

**Figure 1:**
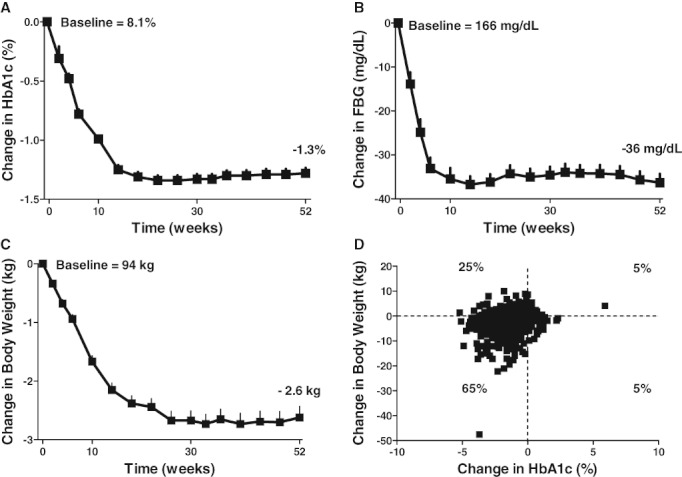
Changes in glycaemic control and body weight over 52 weeks. Patients (Intent-to-Treat, ITT population; N = 675) treated with exenatide once weekly (ExQW) for up to 52 weeks experienced reductions [mean change (SE)] from baseline in (A) HbA1c, (B) fasting blood glucose (FBG), and (C) body weight. (D) The majority of ITT patients experienced reductions in both HbA1c and body weight.

Patients receiving ExQW experienced sustained improvements in HbA1c at Week 52 (mean [SE] change from baseline, −1.3% [0.05], p < 0.0001) (Figure 1A). Significant HbA1c improvements were observed regardless of baseline HbA1c [≥9.0%, −2.3% (0.11), p < 0.0001; <9%, −1.0 (0.04), p < 0.0001]. Of patients in the Completer population, 68% achieved HbA1c < 7.0%, 50% achieved HbA1c ≤ 6.5% and 23% achieved HbA1c ≤ 6.0%. Patients also experienced sustained reductions in fasting blood glucose (FBG) concentrations [−36.3 (2.02) mg/dl, p < 0.0001; Figure 1B].

Changes in background antihyperglycaemia agents were allowed only if deemed necessary by the investigator. For subjects in the Completer population, of 490 patients taking metformin alone or in combination with other antihyperglycaemia therapies at study entry, 97% (477 patients) did not change their dose during the 52-week period. Of the small number of patients who took metformin at baseline and changed their metformin dose (n = 14), five increased and eight decreased their dose. One patient initiated metformin treatment during the 52-week period, who later decreased their metformin dose. Among the 136 patients taking a TZD alone or in combination with other antihyperglycaemia therapies at baseline, 97% (132 patients) did not change their dose, 2 patients increased their dose and 2 patients decreased their dose. No patients initiated TZD therapy during the 52-week period. Of 103 patients taking a SU alone or in combination with other antihyperglycaemia therapies at baseline, 49% (50 patients) did not change their dose, 36% (37 patients) increased their dose and 16% (16 patients) decreased their dose. Six patients initiated SU therapy during the 52-week period, 4 of whom did not alter their regimen and 2 of whom increased their dose.

Mean body weight decreased progressively, with a −2.6 (0.19) kg (p < 0.0001) decrease at Week 52 (Figure 1C). With ExQW treatment, 88% of ITT patients achieved HbA1c reductions and 63% achieved reductions in HbA1c and body weight (Figure 1D). Clinically significant HbA1c reductions were observed regardless of weight loss.

### Effects on Blood Pressure, ALT and Fasting Lipid Concentrations

**Figure 2:**
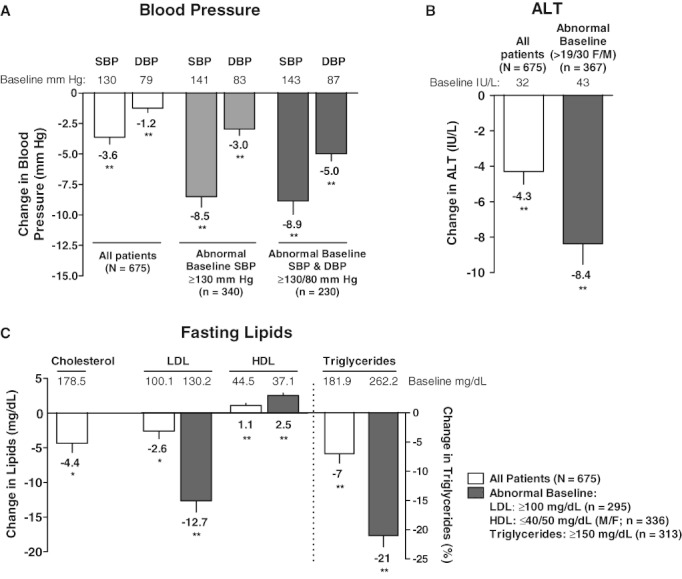
Change in blood pressure, alanine aminotransferase (ALT) and fasting lipids. At Week 52, patients (Intent-to-Treat, ITT population; N = 675) exhibited reductions in (A) systolic (SBP) and diastolic blood pressure (DBP), with greater improvements in patients with abnormal blood pressure at baseline, (B) alanine aminotransferase (ALT) concentrations, with further improvements observed in patients with abnormal ALT levels at baseline, and (C) fasting serum lipids, with greater improvements in patients with abnormal levels at baseline. Data are presented as mean change (SE) from baseline except for triglycerides [geometric mean mg/dl baseline; geometric mean percent change (SE) from baseline]. *p < 0.05, **p < 0.001.

Clinically significant reductions in blood pressure were observed at Week 52 (systolic blood pressure [SBP]: −3.6 (0.56) mm Hg, p < 0.0001; diastolic blood pressure [DBP]: −1.2 (0.34) mm Hg p < 0.001) (Figure 2A). Greater improvements were observed in patients with elevated baseline SBP [≥130 mm Hg): SBP −8.5 (0.85) mm Hg, p < 0.0001; DBP: −3.0 (0.50) mm Hg, p < 0.0001], or elevated baseline SBP and DBP (≥130/80 mm Hg): SBP: −8.9 (1.10) mm Hg, p < 0.0001, DBP: −5.0 (0.58) mm Hg p < 0.0001. Of ITT patients with abnormal SBP at baseline, 41% achieved normal SBP at Week 52.

Changes in antihypertensive medication regimens were allowed only if deemed necessary by the investigator. Of 446 patients taking antihypertensive agents at screening, 85% (379 patients) did not change their dose during the 52-week period. Of the small number who changed their antihypertensive regimen, 46 (10%) increased their dose, 19 (4%) decreased their dose, and 2 (0.4%) discontinued antihypertensive therapy. Twenty-seven patients (4.0%) initiated antihypertensive treatment, 23 (85%) of whom did not alter their regimen and 4 (15%) of whom increased their dose.

ExQW treatment also resulted in clinically significant reductions in the liver injury biomarker ALT at 52 weeks [−4.3 (0.71) IU/l, p < 0.0001] (Figure 2B). Greater reductions (−8.4 [1.17] IU/l, p < 0.0001) were observed in patients with abnormal baseline ALT concentrations (>19 IU/l female, >30 IU/l male), with 34% exhibiting normal values at Week 52.

Although, mean fasting lipid concentrations were within normal limits at baseline, significant improvements were observed in total cholesterol [−4.4 (1.33) mg/dl p = 0.0011], LDL [−2.6 (1.08) mg/dl, p = 0.0158], high-density lipoprotein (HDL) [1.1 (0.31) mg/dl, p = 0.0004] and triglyceride [−7% (1.6), p = 0.0002] concentrations at Week 52 (Figure 2C). Patients with abnormal baseline LDL (≥100 mg), HDL (≤40/50 mg/dl, male/female), or triglyceride (≥150 mg/dl) concentrations exhibited greater improvements in those analytes with ExQW treatment [LDL: −12.7 (1.61) mg/dl, p < 0.0001; HDL: 2.5 (0.34) mg/dl, p < 0.0001; triglycerides: −21% (1.9), p < 0.0001].

Changes in lipid-lowering medications were only allowed if deemed necessary by the investigator. Of 355 patients taking lipid-lowering agents at screening, 90% (321 patients) did not change their dose during the treatment period. Of the small number who did change their lipid-lowering regimen, 15 (4%) increased their dose, 12 (3%) decreased their dose, and 7 (2%) discontinued their lipid-lowering agent. Twenty-two patients (3.3%) initiated treatment with a lipid-lowering medication during the 52-week period.

Improvements in HDL (r = −0.10), triglyceride (r = 0.08), and ALT (r = 0.17) concentrations were modestly but significantly correlated with changes in body weight (p < 0.05). Changes in SBP (r = 0.03, p = 0.48), DBP (r = 0.07, p = 0.06), total cholesterol (r = 0.06, p = 0.15), and LDL (r = 0.05, p = 0.26) concentrations, however, were not significantly correlated with weight loss. Effects of ExQW on glycaemic and cardiometabolic parameters were similar between the ITT and Completer populations.

### Effects of ExQW by Background Antihyperglycaemia Therapy

The effects of ExQW were also assessed by background antihyperglycaemia therapy. Several groups had only a small number of patients (ITT, SU only, n = 6; TZD only, n = 17; diet/exercise only, n = 20), limiting interpretation of results for these groups. Data for all groups in the ITT population are provided in Table S1; however, discussion of the results will focus on groups with larger patient populations (metformin-only, n = 366; metformin + TZD, n = 132; SU + other oral antihyperglycaemia agents, n = 126).

Across background antihyperglycaemia therapies, significant reductions in HbA1c, FBG and body weight were observed. Significant improvements were also observed in SBP, DBP, LDL, HDL and triglyceride concentrations across background antihyperglycaemia treatment, with greater improvements observed in patients with abnormal baseline concentrations. Significant reductions in total cholesterol were observed in patients on backgrounds of metformin + TZD and SU + other oral agents. Significant reductions in ALT concentrations were observed in patients treated with metformin or SU + other oral agents, with greater improvements observed in patients with elevated ALT concentrations at baseline. Results were generally consistent between ITT and Completer populations (Table S2).

### Treatment Goals

**Figure 3:**
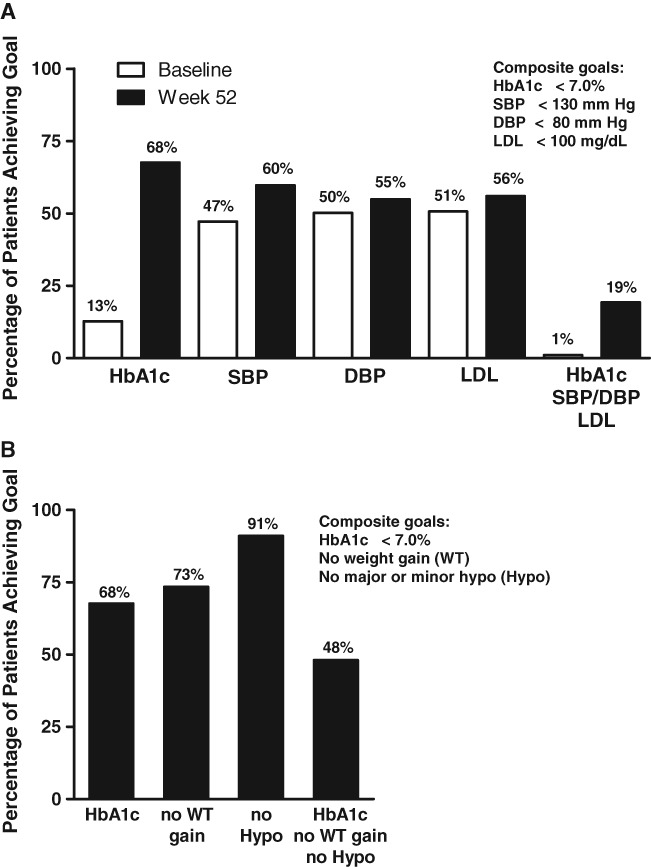
Proportion of patients achieving targets. Exenatide once weekly (ExQW) treatment was associated with increased percentages of patients (52-week Completer population, N = 534) that achieved (A) American Diabetes Association (ADA) goals of HbA1c < 7.0%, systolic/diastolic blood pressure (SBP/DBP) < 130/80 mm Hg, low-density lipoprotein (LDL) < 100 mg/dl, and (B) targets of HbA1c < 7.0%, no weight gain and no major/minor hypoglycaemia.

ExQW treatment increased the percentages of patients achieving goals recommended by the ADA to reduce the risk of complications (HbA1c < 7.0%, blood pressure < 130/80 mm Hg, LDL < 100 mg/dl). The percentage of patients achieving HbA1c < 7.0% increased from 13% at baseline to 68% (Figure 3A). Sixty percent of patients achieved SBP < 130 mm Hg, compared to 47% at baseline; 55% of patients reached DBP < 80 mm Hg versus 50% at baseline. The percentage of patients achieving LDL < 100 mg/dl was 56%, compared to 51% at baseline. While only 1% of patients met the composite goal for HbA1c, SBP/DBP and LDL at baseline, 19% achieved the composite goal following 52 weeks of ExQW treatment.

ExQW also assisted patients in achieving another clinically relevant composite outcome, specifically whether the treatment goal of HbA1c < 7.0% can be achieved without weight gain or increased hypoglycaemia risk [3]. At Week 52, 68% of patients achieved HbA1c < 7.0%, 73% showed no weight gain, and 91% had no episodes of major or minor hypoglycaemia, resulting in 48% achieving the composite of all three goals (Figure 3B).

### Safety and Tolerability

ExQW was generally well tolerated. The most frequent TEAEs were nausea and diarrhoea (Table 2). Hypoglycaemia events were infrequent, with a significantly (p < 0.0001) greater frequency observed with a concomitant SU (Table 3). There was one hypoglycaemic event in a patient not using a concomitant SU that necessitated the assistance of another person but did not involve loss or severe impairment of consciousness.

**Table 2:** Frequent adverse events with an overall incidence ≥10% (Intent-to-Treat population, N = 675)

Preferred term	n (%)
Nausea	144 (21.3)
Diarrhoea	98 (14.5)
Nasopharyngitis	83 (12.3)
Vomiting	69 (10.2)

**Table 3:** Incidence of hypoglycaemia by concomitant sulfonylurea (SU) use (Intent-to-Treat population, N = 675)

	With SU (n = 129)	No SU (n = 546)
	n (%)	n (%)
Hypoglycaemia		
Major hypoglycaemia	0 (0.0)	0 (0.0)
Minor hypoglycaemia	27 (20.9)	18 (3.3)*

* p < 0.0001 between groups (SU vs. no SU) by Fisher's Exact test.

## Discussion

Patients with T2DM have twice the risk of vascular diseases [13] and current treatment recommendations for T2DM emphasize not only glycaemic control but also consideration of cardiometabolic comorbidities. Direct effects of antihyperglycaemic medications on CV risk markers should therefore be considered when individualizing treatment regimens for patients with T2DM.

Accumulating evidence has reported that GLP-1RAs improved not only glycaemic control, but also CV risk factors [14]. In addition to glycaemic improvements, ExBID treatment resulted in clinically relevant reductions in body weight, blood pressure and other CV risk factors in clinical trials [5,10,15,16] with similar improvements reported in real-world settings [17–19]. Another GLP-1RA, liraglutide, has also been reported to elicit reductions in body weight, SBP, HDL, triglycerides and CV risk biomarkers [20].

Multiple assessments also indicated no increased risk of CV outcomes with GLP-1RA treatment. In a pooled retrospective analysis of data from 12 clinical trials, no increased CV risk was associated with ExBID compared to placebo or insulin [21]. Similarly, in a retrospective analysis of a large insurance database, 39 275 patients receiving ExBID were significantly less likely to have a CV event than 381 218 patients treated with other glucose-lowering therapies [22]. In a meta-analysis of clinical trials, liraglutide was associated with low incidence rates of MACE that were similar to rates observed for the comparator arms [23]. In a meta-analysis of MACE events occurring in GLP-1RA trials, GLP-1RAs (ExBID, ExQW, liraglutide and albiglutide) were associated with significant reductions in CV morbidity compared to placebo, although not versus active comparator [24].

In the analysis reported here, 52 weeks of ExQW was associated with clinically relevant improvements in glycaemic control (HbA1c and FBG) and reductions in CV risk factors (obesity, hypertension, dyslipidemia and a hepatic injury biomarker). The majority of patients (63%) experienced reductions in both HbA1c and body weight, and the improvements in SBP, DBP, total cholesterol and LDL concentrations were not statistically correlated with weight loss. These improvements led to 19% of patients achieving the composite ADA goal (HbA1c < 7.0%, BP < 130/80 mm Hg, LDL < 100 mg/dl) following 52 weeks of ExQW, compared to 1% at baseline.

In comparison, an analysis was conducted using data from the National Health and Nutrition Examination Survey (NHANES) to assess diabetes prevalence and therapeutic target achievement for US patients using diet and exercise, oral antihyperglycaemic medications and/or insulin. Data from 2003 to 2006 indicate that only one in eight patients (12.2%) achieved the composite ADA goal [2].

The effects of ExQW reported here were further assessed by background antihyperglycaemic agents. Improvements in glycaemic control and CV risk factors with ExQW therapy were observed across the range of antihyperglycaemic agents commonly used to treat T2DM, with the greatest effect observed in patients with abnormal analyte level at baseline. These results are consistent with those reported previously for ExQW [25].

Furthermore, these improvements are not likely explained by increases in the dose of background antihyperglycaemic therapies, as most patients did not alter their background dose. The exception was the group of patients taking SU, where nearly half increased their SU dose during study conduct. This was primarily because of patients from the DURATION-1 trial, where patients were required to reduce their SU dose to the minimum labelled dose through Week 10 to mitigate the risk of hypoglycaemia [7]. During the remainder of the study period, their SU dose was up-titrated based on daily glucose monitoring to achieve targeted FBG concentrations. Of the 37 patients in this analysis that increased their SU dose during the 52-week period, 36 were enrolled in DURATION-1.

Many current T2DM therapies, while improving glycaemic control, are often associated with further weight gain that may increase CV risk and with hypoglycaemia that may also adversely affect CV risk and patient compliance to therapy. A composite goal has been proposed that relates achievement of HbA1c < 7% with no weight gain and no hypoglycaemia events to compare the clinical effectiveness of antihyperglycaemic therapies and assist physicians in selecting appropriate agents for individualized therapy [3]. In a meta-analysis of the 26-week LEAD trials, 32% of liraglutide 1.2 mg patients and 40% of liraglutide 1.8 mg patients reached this composite goal [3]. In LEAD-6, 25% of ExBID patients achieved the composite goal at Week 26 [3]. In the analysis reported here with 52 weeks of ExQW exposure, nearly half (48%) of the patients achieved the composite goal of HbA1c < 7% with no weight gain and no hypoglycaemia.

An increased incidence of major CV events has been reported in patients with non-alcoholic fatty liver disease (NAFLD) [26,27]. T2DM and obesity are the major risk factors for NAFLD, and reduction of body weight is currently the only recommended therapy [28]. As definitive diagnosis requires histological examination of hepatic tissue, we cannot confirm the presence of NAFLD in any patient by elevated levels of the liver injury biomarker ALT. However, in patients with T2DM, NAFLD is the most common cause of ALT elevation and a reduction in ALT is likely to reflect a decrease in liver inflammation. In this study, we report improvements in ALT with ExQW treatment, with 34% of patients with abnormal baseline ALT values exhibiting normal values at Week 52. The reduction in ALT levels was modestly but significantly correlated with change in body weight.

A limitation of this trial is the pooling of data from controlled (with 24-week uncontrolled extensions) and uncontrolled clinical trials. The open-label portions may have selected patients who responded to and tolerated the treatment well. However, analyses were conducted using the LOCF method in which all data, even from patients who withdrew, were included, thereby providing the most conservative assessment of changes from baseline. In addition, the results presented here showed improvements in glycaemic parameters, body weight, blood pressure and lipid concentrations that are similar to previous reports from the double-blind, comparator-controlled periods [7,8,9,11]. With regard to the change from baseline data assessment, greater changes were observed in patients with abnormal baseline values, which may be partially due to the regression to the mean phenomena and therefore pose a limitation to the data interpretation. Conclusions drawn from this analysis are also limited by the absence of long-term CV outcome measures. A large CV outcomes trial, Exenatide Study of Cardiovascular Event Lowering (EXSCEL), is currently underway to determine the effect of ExQW on the incidence of CV events in patients with T2DM.

## Conclusion

ExQW therapy for 52 weeks resulted in significant improvements in glycaemic control, coupled with clinically relevant reductions in body weight, blood pressure, fasting lipids and hepatic injury biomarker ALT regardless of background antihyperglycaemia therapy. ExQW also increased the likelihood of achieving a clinically relevant composite outcome of an HbA1c < 7% without weight gain or increased risk of major/minor hypoglycaemia, as well as the composite ADA goals of HbA1c < 7%, SBP/DBP < 130/80 mm Hg and LDL < 100 mg/dl. Therefore, ExQW may offer a therapeutic option for patients with T2DM since it has the potential to impact glycaemic control, blood pressure and LDL without increasing body weight or the risk of hypoglycaemia.

## Footnotes

a ClinicalTrials.gov identifiers: NCT00308139, NCT00637273, NCT00641056, NCT00753896.

## Supporting Information

**Table S1.** The effect of exenatide once weekly on glycaemic and cardiometabolic parameters by background oral antihyperglycaemia agents – ITT population (N = 675).

**Table S2.** The effect of exenatide once weekly on glycaemic and cardiometabolic parameters by background oral antihyperglycaemia agents – completer population (N = 534).
