# Supplementary material for: Exenatide once weekly improved glycaemic control, cardiometabolic risk factors and a composite index of an HbA1c < 7%, without weight gain or hypoglycaemia, over 52 weeks
Source: Diabetes Obes Metab. 2012 Nov 12;15(3):264–71. doi: 10.1111/dom.12026 (PMC3593159; doi:10.1111/dom.12026)
Supplement: Supplementary file 1 [file dom0015-0264-SD1.doc]

Supporting information

Table S1. The Effect of Exenatide Once Weekly on Glycaemic and Cardiometabolic Parameters by Background Oral Antihyperglycaemia Agents – ITT Population (N = 675)

|  |  | **Background Antihyperglycaemia Treatment** | | | | | |
| --- | --- | --- | --- | --- | --- | --- | --- |
| **Parameter** |  | **Diet/Exercise Only (n = 20)** | **SU Only (n = 6)** | **TZD Only (n = 17)** | **MET Only (n = 366)** | **MET + TZD (n = 132)** | **SU + Other Oral Agents (n = 126)** |
| **HbA1c (%)** |  |  |  |  |  |  |  |
| Total | Baseline | 8.22 (0.22) | 7.62 (0.32) | 7.29 (0.30) | 8.35 (0.06) | 7.36 (0.09) | 8.48 (0.10) |
|  | Change | -1.31 (0.27)**** | -0.87 (0.31) | -0.96 (0.25)*** | -1.42 (0.07)**** | -0.85 (0.07)**** | -1.42 (0.11)**** |
|  |  |  |  |  |  |  |  |
| <9% | n | 15 | 5 | 15 | 264 | 123 | 85 |
|  | Baseline | 7.74 (0.15) | 7.34 (0.20) | 6.98 (0.24) | 7.77 (0.04) | 7.19 (0.07) | 7.79 (0.06) |
|  | Change | -0.93 (0.25)** | -0.68 (0.30) | -0.67 (0.17)** | -1.07 (0.06)**** | -0.76 (0.07)**** | -0.93 (0.09)**** |
|  |  |  |  |  |  |  |  |
| ≥9% | n | 5 | 1 | 2 | 101 | 9 | 41 |
|  | Baseline | 9.66 (0.20) | 9.00† | 9.60 (0.00) | 9.86 (0.06) | 9.70 (0.10) | 9.92 (0.10) |
|  | Change | -2.44 (0.51)** | -1.8† | -3.15 (0.15)* | -2.31 (0.14)**** | -2.04 (0.40)*** | -2.40 (0.19)**** |
|  |  |  |  |  |  |  |  |
| **FBG (mg/dL)** |  |  |  |  |  |  |  |
| Total | Baseline | 166.6 (11.18) | 158.6 (13.48) | 151.0 (12.01) | 167.7 (2.39) | 149.4 (3.78) | 180.5 (5.37) |
|  | Change | -36.2 (10.05)** | -11.1 (17.6) | -34.2 (8.67)** | -37.8 (2.79)**** | -30.8 (3.65)**** | -42.8 (5.37)**** |
|  |  |  |  |  |  |  |  |
| **Body Weight (kg)** |  |  |  |  |  |  |  |
| Total | Baseline | 105.3 (4.59) | 97.0 (7.32) | 90.7 (4.25) | 92.2 (1.02) | 99.0 (1.62) | 94.1 (1.73) |
|  | Change | -2.8 (1.26)* | -1.1 (1.55) | -0.8 (1.28) | -2.9 (0.24)**** | -1.9 (0.45)**** | -2.7 (0.50)**** |
|  |  |  |  |  |  |  |  |
| **SBP (mm Hg)** |  |  |  |  |  |  |  |
| Total | Baseline | 128.5 (2.85) | 137.5 (6.96) | 126.7 (3.90) | 130.24 (0.80) | 127.5 (1.14) | 131.5 (1.52) |
|  | Change | -0.7 (3.53) | -7.8 (6.16) | -3.2 (3.45) | -3.6 (0.76)**** | -3.7 (1.11)** | -4.1 (1.42)** |
|  |  |  |  |  |  |  |  |
| Abnormal SBP at baseline | n | 13 | 4 | 9 | 182 | 63 | 66 |
|  | Baseline | 136.1 (2.14) | 144.8 (8.26) | 139.3 (2.65) | 141.9 (0.94) | 138.0 (1.18) | 144.1 (1.64) |
|  | Change | -7.5 (3.76) | -12.3 (6.86) | -7.0 (5.40) | -7.8 (1.18)**** | -9.3 (1.65)**** | -9.9 (2.25)**** |
|  |  |  |  |  |  |  |  |
| Abnormal SBP/DBP at baseline | n | 15 | 5 | 10 | 251 | 84 | 81 |
|  | Baseline | 133.7 (2.47) | 140.2 (7.85) | 137.2 (3.19) | 136.1 (0.92) | 134.0 (1.20) | 139.8 (1.69) |
|  | Change | -3.2 (4.38) | -12.2 (5.31) | -7.7 (4.88) | -5.0 (0.99)**** | -6.9 (1.40)**** | -8.0 (1.96)**** |
|  |  |  |  |  |  |  |  |
| **DBP (mm Hg)** |  |  |  |  |  |  |  |
| Total | Baseline | 79.7 (1.33) | 77.8 (0.91) | 72.9 (2.00) | 79.9 (0.50) | 76.5 (0.65) | 78.1 (0.86) |
|  | Change | 0.4 (2.04) | -4.3 (2.54) | -1.8 (2.26) | -1.7 (0.47)*** | -0.7 (0.70) | -0.7 (0.80) |
|  |  |  |  |  |  |  |  |
| Abnormal SBP at baseline | n | 13 | 4 | 9 | 182 | 63 | 66 |
|  | Baseline | 81.9 (1.45) | 78.3 (0.63) | 76.8 (1.84) | 84.0 (0.70) | 79.2 (0.83) | 82.6 (1.03) |
|  | Change | -0.5 (2.70) | -4.3 (3.97) | -5.8 (2.81) | -3.3 (0.72)**** | -3.2 (0.94)** | -2.1 (1.19) |
|  |  |  |  |  |  |  |  |
| Abnormal SBP/DBP at baseline | n | 15 | 5 | 10 | 251 | 84 | 81 |
|  | Baseline | 82.0 (1.27) | 78.6 (0.60) | 77.1 (1.68) | 83.8 (0.52) | 80.1 (0.66) | 82.7 (0.86) |
|  | Change | 0.2 (2.37) | -4.6 (3.09) | -5.8 (2.52)* | -3.4 (0.57)**** | -3.2 (0.77)**** | -2.6 (1.01)* |
|  |  |  |  |  |  |  |  |
| **Total cholesterol (mg/dL)** | Baseline | 168.2 (8.10) | 176.7 (30.08) | 193.5 (15.02) | 177.1 (2.10) | 183.1 (3.95) | 176.1 (3.87) |
|  | Change | 1.4 (8.08) | 3.6 (9.65) | -12.5 (13.14) | -1.4 (1.74) | -10.0 (3.19)** | -5.4 (2.64)* |
|  |  |  |  |  |  |  |  |
| **LDL (mg/dL)** |  |  |  |  |  |  |  |
| Total | Baseline | 97.1 (6.53) | 71.4 (17.20) | 103.7 (10.69) | 100.7 (1.80) | 103.6 (3.32) | 95.5 (3.26) |
|  | Change | -0.9 (4.78) | 12.9 (12.87) | -5.3 (9.85) | -0.6 (1.43) | -7.9 (2.48)** | -2.5 (2.32) |
|  |  |  |  |  |  |  |  |
| Abnormal at baseline | n | 8 | 2 | 8 | 160 | 61 | 51 |
|  | Baseline | 125.7 (7.03) | 118.5 (0.39) | 137.1 (14.74) | 128.2 (1.91) | 136.7 (3.19) | 128.2 (3.27) |
|  | Change | 1.1 (10.25) | -19.9 (2.12) | -25.1 (13.21) | -9.3 (1.93)**** | -18.3 (3.92)**** | -15.4 (3.80)*** |
|  |  |  |  |  |  |  |  |
| **HDL (mg/dL)** |  |  |  |  |  |  |  |
| Total | Baseline | 41.6 (2.45) | 45.2 (4.79) | 50.4 (2.69) | 43.6 (0.60) | 46.4 (1.20) | 44.3 (0.96) |
|  | Change | 4.2 (1.82)* | -0.3 (1.88) | -1.5 (1.97) | 1.0 (0.39)** | 2.1 (0.70)** | 0.7 (0.77) |
|  |  |  |  |  |  |  |  |
| Abnormal at baseline | n | 11 | 3 | 6 | 187 | 61 | 64 |
|  | Baseline | 34.6 (1.41) | 37.7 (5.77) | 40.5 (2.58) | 36.9 (0.43) | 36.6 (0.79) | 38.6 (0.82) |
|  | Change | 6.9 (2.69)* | -0.4 (3.59) | 4.1 (3.05)* | 2.3 (0.42)**** | 3.7 (0.75)**** | 1.3 (0.94) |
|  |  |  |  |  |  |  |  |
| **Triglycerides** |  |  |  |  |  |  |  |
| Total | Baseline (mg/dL) | 154.8 (22.14) | 349.1 (179.51) | 197.7 (53.80) | 180.6 (6.12) | 168.4 (8.70) | 195.2 (11.88) |
|  | Change (%) | -10 (8.1) | -6 (18.5) | 4 (15.3) | -4 (2.2) | -11 (3.4)** | -10 (3.6)** |
|  |  |  |  |  |  |  |  |
| Abnormal at baseline | n | 7 | 4 | 7 | 174 | 53 | 64 |
|  | Baseline | 256.1 (38.87) | 462.2 (260.33) | 370.0 (100.65) | 253.9 (9.33) | 254.8 (13.72) | 268.5 (17.56) |
|  | Change | -16 (14.7) | -20 (20.6) | -32 (7.6) | -17 (2.6)*** | -30 (4.3)**** | -25 (4.2)*** |
|  |  |  |  |  |  |  |  |
| **ALT (mg/dL)** |  |  |  |  |  |  |  |
| Total | Baseline | 38.8 (3.60) | 35.0 (11.64) | 23.9 (2.59) | 33.8 (1.11) | 25.7 (1.15) | 34.0 (1.74) |
|  | Change | -11.7 (3.37)** | 2.2 (2.30) | -3.0 (4.44) | -4.4 (1.00)**** | -1.3 (1.15) | -5.69 (1.66)*** |
|  |  |  |  |  |  |  |  |
| Abnormal at baseline | n | 12 | 3 | 5 | 216 | 50 | 77 |
|  | Baseline | 49.4 (3.02) | 52.0 (19.50) | 33.2 (5.06) | 44.0 (1.51) | 35.8 (2.18) | 42.7 (2.31) |
|  | Change | -18.3 (4.14)** | -0.3 (3.84) | -6.8 (6.87) | -8.0 (1.61)**** | -4.8 (2.58) | -9.6 (2.49)*** |
| Abbreviations: MET = metformin, SU = sulfonylurea, SU + Other Oral Agents = SU + metformin and/or TZD; TZD = thiazolidinedione.  Notes: - Eight patients received insulin treatment during hospitalizations for adverse events and were excluded from this analysis.   - Values represent mean ± SE.   ****<0.0001  ***<.001  ** < .01  * <.05  † n = 1 for this group. No SE calculated. | | | | | | | |

Table S2. The Effect of Exenatide Once Weekly on Glycaemic and Cardiometabolic Parameters by Background Oral Antihyperglycaemia Agents - Completer Population (N = 534)

|  |  | **Background Antihyperglycaemia Treatment** | | | | | |
| --- | --- | --- | --- | --- | --- | --- | --- |
| **Parameter** |  | **Diet/Exercise Only (n = 17)** | **SU Only (n = 5)** | **TZD Only (n = 16)** | **MET Only (n = 273)** | **MET + TZD (n = 115)** | **SU + Other Oral Agents (n = 104)** |
| **HbA1c (%)** |  |  |  |  |  |  |  |
| Total | Baseline | 8.27 (0.24) | 7.74 (0.37) | 7.37 (0.31) | 8.31 (0.07) | 7.31 (0.09) | 8.43 (0.11) |
|  | Change | -1.53 (0.26)**** | -1.06 (0.29)* | -1.03 (0.25)*** | -1.48 (0.08)**** | -0.84 (0.08)**** | -1.52 (0.11)**** |
|  |  |  |  |  |  |  |  |
| <9% | n | 13 | 4 | 14 | 205 | 109 | 70 |
|  | Baseline | 7.82 (0.15) | 7.43 (0.24) | 7.05 (0.25) | 7.80 (0.04) | 7.18 (0.07) | 7.73 (0.07) |
|  | Change | -1.13 (-0.22)*** | -0.88 (0.29) | -0.73 (0.17)*** | -1.12 (0.07)**** | -0.79 (0.07)**** | -1.03 (0.08)**** |
|  |  |  |  |  |  |  |  |
| ≥9% | n | 4 | 1 | 2 | 68 | 6 | 34 |
|  | Baseline | 9.73 (0.25) | 9.00 (n/a)† | 9.60 (0.00) | 9.87 (0.08) | 9.70 (0.15) | 9.86 (0.12) |
|  | Change | -2.83 (0.44)** | -1.80 (n/a)† | -3.15 (0.15)* | -2.55 (0.16)**** | -1.82 (0.47)* | -2.53 (0.20)**** |
|  |  |  |  |  |  |  |  |
| **FBG (mg/dL)** |  |  |  |  |  |  |  |
| Total | Baseline | 166.4 (12.64) | 159.6 (16.45) | 154.2 (12.35) | 166.1 (2.56) | 149.9 (4.03) | 177.7 (5.84) |
|  | Change | -42.4 (9.68)*** | -7.9 (21.20) | -34.2 (8.67)**** | -38.5 (3.18)**** | -31.5 (3.98)**** | -45.1 (5.74)**** |
|  |  |  |  |  |  |  |  |
| **Body Weight (kg)** |  |  |  |  |  |  |  |
| Total | Baseline | 105.8 (5.13) | 96.7 (8.96) | 91.3 (4.48) | 93.0 (1.17) | 100.3 (1.75) | 94.2 (1.92) |
|  | Change | -3.0 (1.36)* | -1.2 (1.89) | -0.7 (1.36) | -3.3 (0.28)**** | -1.9 (0.51)*** | -2.8 (0.56)**** |
|  |  |  |  |  |  |  |  |
| **SBP (mm Hg)** |  |  |  |  |  |  |  |
| Total | Baseline | 128.8 (3.23) | 138.6 (8.41) | 126.8 (4.14) | 130.3 (0.89) | 128.5 (1.24) | 131.4 (1.65) |
|  | Change | 0.0 (4.07) | -9.0 (7.40) | -4.4 (3.45) | -3.2 (0.87)*** | -3.7 (1.21) | -5.8 (1.49) |
|  |  |  |  |  |  |  |  |
| Abnormal SBP at baseline | n | 11 | 3 | 9 | 143 | 60 | 55 |
|  | Baseline | 136.6 (2.50) | 149.0 (10.02) | 139.3 (2.65) | 140.8 (1.01) | 138.2 (1.23) | 143.6 (1.79) |
|  | Change | -7.3 (4.42) | -15.7 (8.41) | -7.0 (5.40) | -7.5 (1.26)**** | -9.5 (1.72)**** | -12.3 (2.20)**** |
|  |  |  |  |  |  |  |  |
| Abnormal SBP/DBP at baseline | n | 13 | 4 | 10 | 188 | 77 | 67 |
|  | Baseline | 133.8 (2.86) | 142.3 (9.78) | 137.2 (3.19) | 136.1 (1.01) | 134.7 (1.27) | 139.5 (1.83) |
|  | Change | -2.3 (5.01) | -14.8 (6.02) | -7.7 (4.88) | -4.8 (1.13)**** | -7.0 (1.51)**** | -10.3 (1.97)**** |
|  |  |  |  |  |  |  |  |
| **DBP (mm Hg)** |  |  |  |  |  |  |  |
| Total | Baseline | 80.5 (1.35) | 77.4 (0.98) | 73.5 (2.03) | 79.7 (0.57) | 76.9 (0.66) | 77.7 (0.93) |
|  | Change | -0.8 (2.11) | -4.4 (3.11) | -2.8 (2.13) | -1.4 (0.56)* | -0.8 (0.73) | -1.2 (0.81) |
|  |  |  |  |  |  |  |  |
| Abnormal SBP at baseline | n | 11 | 3 | 9 | 143 | 60 | 55 |
|  | Baseline | 82.8 (1.25) | 77.7 (0.33) | 76.8 (1.84) | 83.9 (0.77) | 79.1 (0.85) | 82.2 (1.11) |
|  | Change | -2.4 (2.55) | -4.3 (9.71) | -5.8 (2.81) | -3.2 (0.84)*** | -2.9 (0.96)** | -2.7 (1.15)* |
|  |  |  |  |  |  |  |  |
| Abnormal SBP/DBP at baseline | n | 13 | 4 | 10 | 188 | 77 | 67 |
|  | Baseline | 82.8 (1.09) | 78.3 (0.63) | 77.1 (1.68) | 83.7 (0.60) | 79.8 (0.69) | 82.1 (0.92) |
|  | Change | -1.3 (2.26) | -4.8 (3.99) | -5.8 (2.52)* | -3.2 (0.68)**** | -3.0 (0.78)*** | -3.3 (0.98)** |
|  |  |  |  |  |  |  |  |
| **Total cholesterol (mg/dL)** | Baseline | 169.4 (7.15) | 151.0 (19.22) | 195.2 (15.90) | 178.3 (2.45) | 184.1 (4.32) | 174.1 (4.26) |
|  | Change | -5.9 (7.09) | 6.6 (11.26) | -12.5 (13.14)( | -1.3 (2.04) | -10.2 (3.60)** | -4.9 (2.95) |
|  |  |  |  |  |  |  |  |
| **LDL (mg/dL)** |  |  |  |  |  |  |  |
| Total | Baseline | 97.1 (6.44) | 72.9 (21.0) | 104.6 (11.34) | 101.2 (2.12) | 104.3 (3.55) | 93.7 (3.54) |
|  | Change | -3.9 (4.85) | 5.6 (12.91) | -5.3 (9.85) | -0.7 (1.62) | -8.1 (2.79) | -2.3 (2.58) |
|  |  |  |  |  |  |  |  |
| Abnormal at baseline | n | 6 | 2 | 8 | 119 | 52 | 42 |
|  | Baseline | 126.5 (8.47) | 118.5 (0.39) | 137.1 (14.74) | 129.3 (2.21) | 137.6 (3.61) | 126.1 (3.54) |
|  | Change | -3.3 (10.99) | -19.9 (2.12) | -25.1 (13.21) | -9.9 (2.09)**** | -18.9 (4.54)**** | -14.9 (4.29)** |
|  |  |  |  |  |  |  |  |
| **HDL (mg/dL)** |  |  |  |  |  |  |  |
| Total | Baseline | 42.3 (2.82) | 44.1 (5.70) | 49.4 (2.67) | 43.5 (0.68) | 46.2 (1.22) | 44.6 (1.08) |
|  | Change | 2.4 (1.22) | -0.9 (2.15) | -1.5 (1.97) | 1.5 (0.46)** | 2.3 (0.76)** | 0.4 (0.87) |
|  |  |  |  |  |  |  |  |
| Abnormal at baseline | n | 8 | 3 | 6 | 144 | 50 | 50 |
|  | Baseline | 33.4 (1.44) | 37.7 (5.77) | 40.5 (2.58) | 36.9 (0.48) | 36.9 (0.90) | 38.7 (0.95) |
|  | Change | 3.9 (1.00)** | -0.4 (3.59) | 4.1 (3.05) | 2.4 (0.47)**** | 4.1 (0.85)**** | 0.8 (1.07) |
|  |  |  |  |  |  |  |  |
| **Triglycerides (mg/dL)** |  |  |  |  |  |  |  |
| Total | Baseline | 149.9 (18.31) | 170.8 (25.32) | 206.5 (56.49) | 184.5 (7.27) | 169.1 (8.54) | 192.0 (13.42) |
|  | Change (%) | -17 (6.2) | 4 (21.5) | 4 (15.3) | -0.5 (2.5) | -11 (3.8)** | -9 (4.2)* |
|  |  |  |  |  |  |  |  |
| Abnormal at baseline | n | 6 | 3 | 7 | 130 | 47 | 50 |
|  | Baseline | 227.3 (30.83) | 202.7 (29.18) | 370.0 (100.65) | 260.0 (10.86) | 250.2 (12.37) | 270.2 (21.18) |
|  | Change (%) | -20 (15.6) | -11 (29.5) | -32 (7.6) | -16 (3.0)** | -31 (4.6)**** | -24 (4.9)** |
|  |  |  |  |  |  |  |  |
| **ALT (mg/dL)** |  |  |  |  |  |  |  |
| Total | Baseline | 39.2 (4.00) | 35.6 (14.24) | 24.9 (2.57) | 33.9 (1.27) | 25.9 (1.28) | 34.1 (1.91) |
|  | Change | -10.9 (3.66)** | 1.8 (2.78) | -3.0 (4.44) | -4.23 (1.25)*** | -1.1 (1.32) | -7.1 (1.79)**** |
|  |  |  |  |  |  |  |  |
| Abnormal at baseline | n | 10 | 2 | 5 | 170 | 42 | 65 |
|  | Baseline | 50.2 (3.54) | 62.0 (29.00) | 33.2 (5.06) | 43.0 (1.67) | 36.9 (2.50) | 42.3 (2.53) |
|  | Change | -18.2 (4.85)** | -2.5 (5.50) | -6.8 (6.87) | -7.8 (1.91)**** | -5.0 (3.11) | -11.8 (2.60)**** |
| Abbreviations: MET = metformin, SU = sulfonylurea, SU + Other Oral Agents = SU + metformin and/or TZD; TZD = thiazolidinedione.  Notes: - Four patients received insulin treatment during hospitalizations for adverse events and were excluded from this analysis.   - Values represent mean ± SE.   ****<0.0001  ***<.001  ** < .01  * <.05  † n = 1 for this group. No SE calculated. | | | | | | | |
